# The Effect of Temperature on Kinetics and Diffusion Coefficients of Metallocene Derivatives in Polyol-Based Deep Eutectic Solvents

**DOI:** 10.1371/journal.pone.0144235

**Published:** 2015-12-07

**Authors:** Laleh Bahadori, Mohammed Harun Chakrabarti, Ninie Suhana Abdul Manan, Mohd Ali Hashim, Farouq Sabri Mjalli, Inas Muen AlNashef, Nigel Brandon

**Affiliations:** 1 Department of Chemical Engineering, Faculty of Engineering, University of Malaya, Kuala Lumpur, 50603, Malaysia; 2 Department of Earth Science and Engineering, Imperial College London, South Kensington, London, SW7 2AZ, United Kingdom; 3 Department of Chemistry, Faculty of Science, University of Malaya, Kuala Lumpur, 50603, Malaysia; 4 Petroleum & Chemical Engineering Department, Sultan Qaboos University, Muscat, 123, Oman; 5 Department of Chemical and Environmental Engineering, Masdar Institute of Science and Technology, Abu Dhabi, PO Box 54224, United Arab Emirates; Queen's University Belfast, UNITED KINGDOM

## Abstract

The temperature dependence of the density, dynamic viscosity and ionic conductivity of several deep eutectic solvents (DESs) containing ammonium-based salts and hydrogen bond donvnors (polyol type) are investigated. The temperature-dependent electrolyte viscosity as a function of molar conductivity is correlated by means of Walden’s rule. The oxidation of ferrocene (Fc/Fc^+^) and reduction of cobaltocenium (Cc^+^/Cc) at different temperatures are studied by cyclic voltammetry and potential-step chronoamperometry in DESs. For most DESs, chronoamperometric transients are demonstrated to fit an Arrhenius-type relation to give activation energies for the diffusion of redox couples at different temperatures. The temperature dependence of the measured conductivities of DES1 and DES2 are better correlated with the Vogel-Tamman-Fulcher equation. The kinetics of the Fc/Fc^+^ and Cc^+^/Cc electrochemical systems have been investigated over a temperature range from 298 to 338 K. The heterogeneous electron transfer rate constant is then calculated at different temperatures by means of a logarithmic analysis. The glycerol-based DES (DES5) appears suitable for further testing in electrochemical energy storage devices.

## Introduction

In the probe for new types of green solvents, deep eutectic solvents (DESs) as alternative media to ionic liquids (ILs), have received recent attention [1–3]. DESs share many notable properties of conventional ILs such as inherent conductivity, negligible vapor pressure, wide electrochemical potential windows, high thermal stability, non-flammability and outstanding solvation potential [4–6]. When their constituents are chosen wisely, DESs are environmentally friendly, biodegradable, and non-toxic. In addition, DESs can be prepared simply at low cost [7–9]. A variety of practical industrial applications of DESs have already been reported, e.g., as solvents for electrodeposition [10–12], catalysts or solvents for a range of chemical and enzymatic reactions [13,14], solvents for extractions [15–17] and co-solvents in inorganic[18] and organic [19,20] syntheses, as well as in bio-catalysis [21–23]. The most common form of DESs are those composed of quaternary salts (ammonium or phosphonium) with an organic compound (such as an alcohol, acid, amine or amide) which is a hydrogen bond donor (HBD) able to form a bond with the anion of the salt. Some DESs also consist of metal halides or hydrated halides that form a complex with ammonium or phosphonium salts. DESs melt and freeze at lower temperatures than the constituting components [24–29].

Ferrocene/ferrocenium (Fc/Fc^+^) or cobaltocenium/cobaltocene (Cc^+^/Cc) redox couples—common metallocenes—are reversible in most non-aqueous solutions and have been investigated as candidates for internal references in various ILs [30–34]. Quasi-reference electrodes (QREs) with electrochemically reversible couples, such as Fc/Fc^+^ or Cc^+^/Cc have also been used for the development of reference potential scales in ILs [35,36]. The electrochemical oxidation of ferrocene to the mono-cation ferrocenium, has been investigated in numerous organic solvents at different temperatures. Crooks and Bard [37] measured electrochemical parameters of the Fc/Fc^+^ in acetonitrile at a temperature range of 298 to 573 K, including supercritical conditions. Tsierkezos [38] investigated the voltammetric behavior of the Fc/Fc^+^ couple over a range of temperatures in eight different organic solvents. Wang *et al*. [39] used voltammetric data to infer diffusion coefficients of both Fc and Fc^+^ as a function of temperature in acetonitrile systems. Despite all this, there are few reports on the diffusions and kinetics for the Fc/Fc^+^ couple in ILs. Rogers *et al*. [33] examined in detail the voltammetry of the Fc/Fc^+^ and Cc^+^/Cc couples over a wide range of temperatures and concentrations in several ILs. Matsumiya *et al*. [40] studied the temperature dependencies of the diffusion coefficients and rate kinetics of Fc/Fc^+^ in the quaternary ammonium cation and bis(trifluoromethanesulfone) imide anion type ionic liquid using an I^−^/I^3−^ reference electrode. Weaver *et al*. [41] investigated the electrochemistry of ferrocene-functionalized phosphonium ILs at a temperature range of 289 to 353 K. Compton *et al*. [42] reported that the diffusion coefficients of Fc in a pure IL is more temperature dependent than that observed in a CO_2_-saturated IL, therefore the activation energy of diffusion of Fc in the IL was approximately halved when the liquid was saturated with CO_2_. There was concern, however, regarding the fact that the Fc/Fc^+^ process displayed irregular electrochemical behavior in some ILs at different temperatures [42]. In fact, Guo *et al*. [43] reported that Fc molecules could diffuse faster even at lower temperatures if ILs were saturated with CO_2_ at elevated pressures beyond approximately 3 MPa. Taylor *et al*. [44] determined the diffusion coefficients for Fc derivates in five ILs as a function of temperature and the data revealed a disobedience to the Stokes–Einstein equation. However, no systematic study is available on the effects of varying temperatures on the Fc/Fc^+^ oxidation and Cc^+^/Cc reduction processes in DESs.

A DES performs the dual role of electrolyte and solvent and hence represents a different kind of medium for electrochemical applications. Herein, we report electrochemical data obtained by means of cyclic voltammetry and chronoamperometry using DESs based on quaternary ammonium salts and polyol hydrogen bond donors over a range of temperatures. Also the effect of temperature on the physicochemical properties of the studied DESs is examined.

## Materials and Method

### Preparation and characterization of DESs

In this work, 6 DESs based on two ammonium salts were prepared. Choline chloride (C_5_H_14_ClNO) and N,N-diethylenethanol ammonium chloride (C_6_H_16_ClNO) as quaternary ammonium salts as well as triethylene glycol (C_6_H_14_O_4_), glycerol (C_3_H_8_O_3_) and ethylene glycol (C_2_H_6_O_2_) as hydrogen bond donors were obtained from Merck Chemicals (Germany) with high purity (≥ 98%) and were used as-received. All starting chemicals were stored in an inert glove box (Innovative Technology, Pure LabHE, USA) purged with argon (oxygen-free). The water mass fraction of the chemicals (as per the manufacturer's guide) was <10^−3^%. Table 1 lists the DESs studied in the present work. The DESs were formed according to the preparation method described earlier [8–11]. In brief, two components were mixed in the argon-filled glove box (having oxygen and water contents lower than 1 ppm), in the proportions indicated in Table 1, at 348 K, 300 rpm agitation and atmospheric pressure until a homogeneous, colorless liquid formed. The time of the mixing process was about 3–5 h. The densities of the synthesized DESs at various temperatures were determined using a DMA 4100 Density Meter (Anton Paar, Austria) with three replicates for each reading (having an uncertainty of ± 0.00008 g•cm^-3^). The density of water (degassed and distilled) was measured at 298 K and compared with the corresponding values in density tables, to adjust the density meter for accuracy. The results exhibited a difference of ± 0.00005 g•cm^-3^ which showed good accuracy.

**Table 1:** Physicochemical properties of polyol-based DESs.

DESs	Formulae		Molar ratio	*M*_*w*_ (g mol^-1^)	*ρ* (g cm^-3^)	*η* (mPa s)	*σ* (mS cm^-1^)
	Salt	HBD^a^					
DES1	C_5_H_14_ClNO(Choline chloride)	C_6_H_14_O_4_(Triethylene glycol)	1:3	147.52	1.28	164	1.78
DES2	C_5_H_14_ClNO(Choline chloride)	C_3_H_8_O_3_(Glycerol)	1:2	107.93	1.19	322^b^	0.65 ^b^
DES3	C_5_H_14_ClNO(Choline chloride)	C_2_H_6_O_2_(Ethylene glycol)	1:2	87.92	1.11	66 ^b^	5.26 ^b^
DES4	(C_2_H_5_)_2_NCH_2_CH_2_OH.HCl (N,N-diethylenethanol ammonium chloride)	C_6_H_14_O_4_(Triethylene glycol)	1:3	151.03	1.25	229	1.24
DES5	(C_2_H_5_)_2_NCH_2_CH_2_OH.HCl (N,N-diethylenethanol ammonium chloride)	C_3_H_8_O_3_(Glycerol)	1:2	112.60	1.17	577 ^b^	0.25 ^b^
DES6	(C_2_H_5_)_2_NCH_2_CH_2_OH.HCl (N,N-diethylenethanol ammonium chloride)	C_2_H_6_O_2_(Ethylene glycol)	1:2	92.59	1.10	58 ^b^	5.72 ^b^

^a^HBD = Hydrogen Bond Donor

^**b**^data from ref. ^44^

The viscosities of the DESs were obtained by averaging at least three to five measurements, using a Brookfield DV-II + Pro EXTRA instrument. The uncertainty in viscosity measurements did not exceed ±1% of the measured values in this study. The conductivities were determined using a DZS-708 Multi-parameter analyzer, which was calibrated using a 0.001 M standard solution of KCl (Merck).

### Electrochemical measurements

The electrochemical experiments of all Fc and Cc^+^ solutions were studied in a standard three-compartment glass cell, consisting of a 3 mm diameter Glassy Carbon (GC) or 20 μm diameter platinum working electrode, while Ag wire (pre-treated as described in the literature) [45] and Pt. wire were used as the quasi-reference and counter electrodes, respectively. Working electrodes were carefully polished with 0.3 μm alumina paste (Wirth Buehler) and ultrasonically rinsed in acetone. All electrochemical measurements were carried out using a computer-controlled í-Autolab potentiostat (PGSTAT302N). A Faraday cage was also employed to minimize electrochemical noise, which in turn was placed in the argon-filled glove box. The anodic and cathodic limits of each DES were arbitrarily specified as the potential at which the current density reached higher than 0.2 mA cm^-2^.

## Results and Discussion

### Synthesis and physicochemical properties of DESs

DESs prepared by mixing two different quaternary ammonium salts with different polyol HBDs (that have the ability to form a complex with the halide anions of the quaternary ammonium salts) [45] and their fundamental physicochemical properties including density, viscosity and conductivity were examined. The results are reported in Table 1.

**Fig 1:**
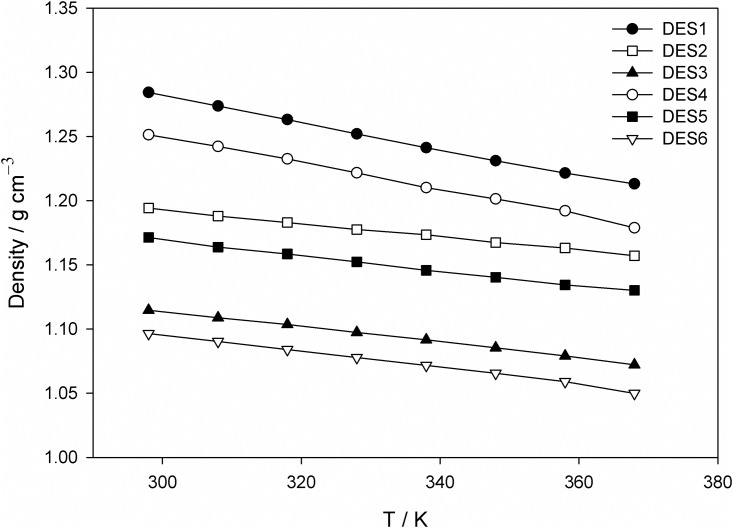
Dependence of densities (*ρ*) on temperature for polyol based DESs.

The densities of DESs were measured over the temperature range of 298 to 368 K at atmospheric pressure. The difference in density could be attributed to a different molecular arrangement or packing of the DES. For the DESs, the density was found to decrease linearly with temperature as shown in Fig 1. Such results would be expected since, as temperature increases, substances (at constant pressure) become less dense due to thermal expansion. The density values were compared to those reported in the literature [46–48] and found to be in good agreement. The following equation fits the experimental data for the densities (*ρ*) of the DESs very well over the entire temperature range:

ρ=AT+B (1)

Where *T* is the absolute temperature while *A* and *B* are empirical constants that depend on the type of DES. As displayed in Table 1, DES1 has a higher density than its other counter parts due to a higher intermolecular packing of the compound’s denser structure. The density of ethylene glycol based DESs were found to be slightly less than those of other polyol-based counterparts.

**Fig 2:**
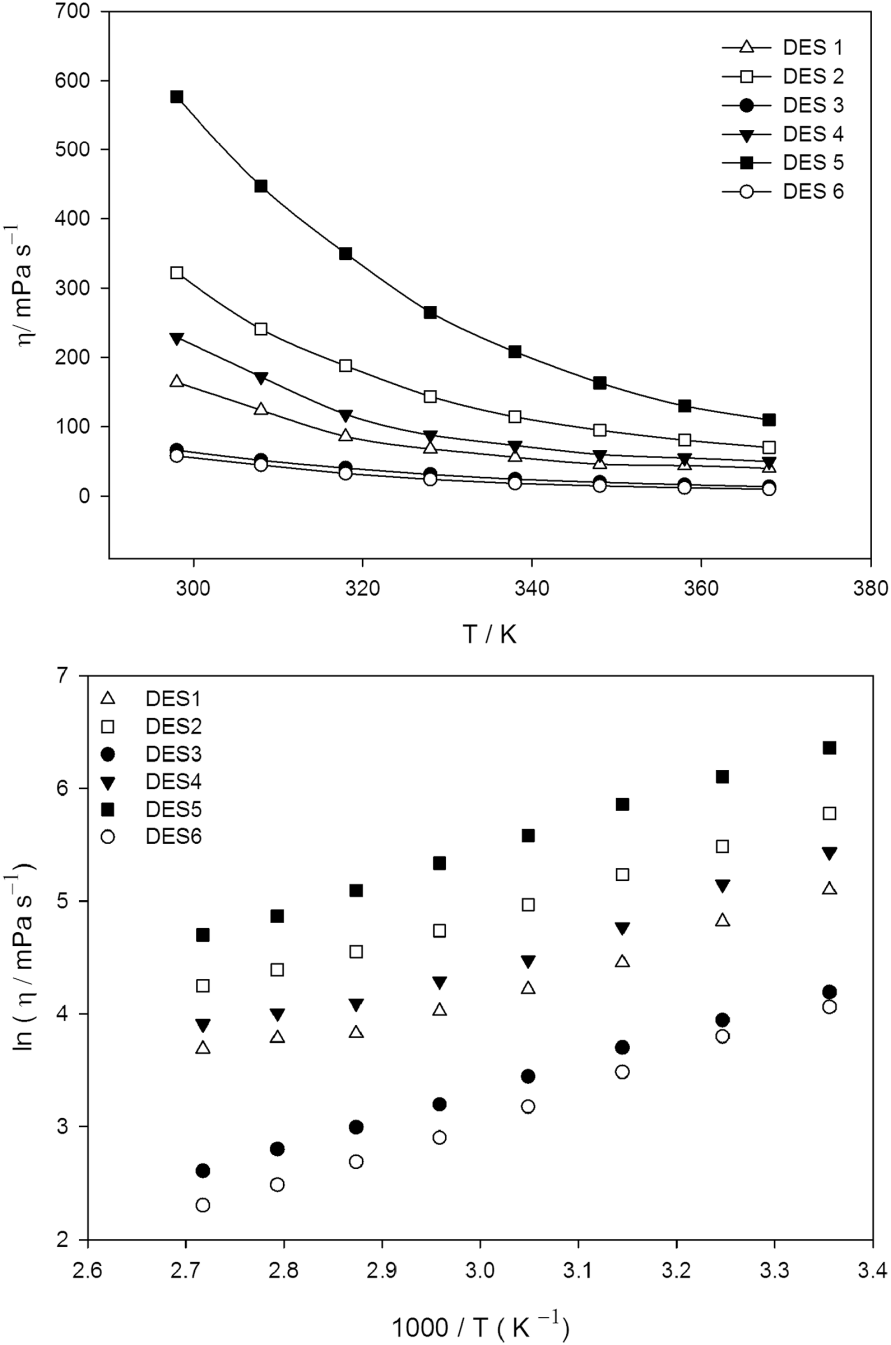
Dynamic viscosity (*η*) of polyol based DESs as a function of temperature; (b) Arrhenius plot of viscosity (*η*) for the polyol based DESs.

The viscosity is a very important parameter in electrochemical studies due to its strong effect on the rate of mass transport within the solution. It significantly influences the diffusion of species, which are dissolved or dispersed in an ionic liquid. The viscosity can be influenced by various parameters such as the relative capacity to form hydrogen bonds, anionic species, size, higher alkalinity, van der Waals forces and cation size [49]. The viscosity is generally affected by the interaction of the salt with the HBD, and their ability to coordinate. Fig 2(a) indicates that, as the temperature increases from 298 to 368 K, the viscosity of the DESs decrease due to the higher mobility of ions. DESs with the glycerol HBD exhibits higher viscosities than other polyol based DESs, resulting in lower conductivities. Moreover, choline chloride based DESs [50] show lower viscosities in comparison to diethylenethanol ammonium chloride based DESs, and the value at 298 K follows the order: **DES5 > DES2 > DES4 > DES1 > DES3 > DES6**.

The temperature dependency of the viscosity (*η*) for the DESs and the profiles are fitted to the Arrhenius equation (Eq (2)), which describes the temperature dependence for non-associating electrolytes [51], as depicted in Fig 2(b).

$$\text{ln}\eta =\text{ln}\eta_{{}_{0}}+\frac{E_{\eta }}{RT}$$ (2)

Where *T* is the temperature in Kelvin, *η* is the viscosity, *E*_*η*_ is the activation energy, *η*_*0*_ is a constant and *R* is the universal gas constant. The value of *E*_*η*_, *η*_*0*_ and sums of square errors are tabulated in Table 2. The regression correlation coefficients have values higher than 0.99 showing a reasonably good fit.

**Table 2:** Regression Parameters for viscosity and conductivity of polyol-based DESs.

DESs	*η*_*0*_/ mPa s	*E*_*η*_/ kJ mol^-1^	SS*E*_*η*_	*σ*_*o*_/mS cm^-1^	*E*_*σ*_/kJ mol^-1^	SS*E*_*σ*_
DES1	1.85	20.68	0.036	10.02	23.97	0.068
DES2	2.59	22.55	0.066	15.44	38.48	0.197
DES3	4.47	20.46	0.035	10.56	21.61	0.097
DES4	1.08	21.30	0.028	11.13	25.70	0.102
DES5	2.80	23.96	0.034	16.03	40.72	0.160
DES6	5.69	19.00	0.054	8.88	17.52	0.046

SS = Sum of Squares

**Fig 3:**
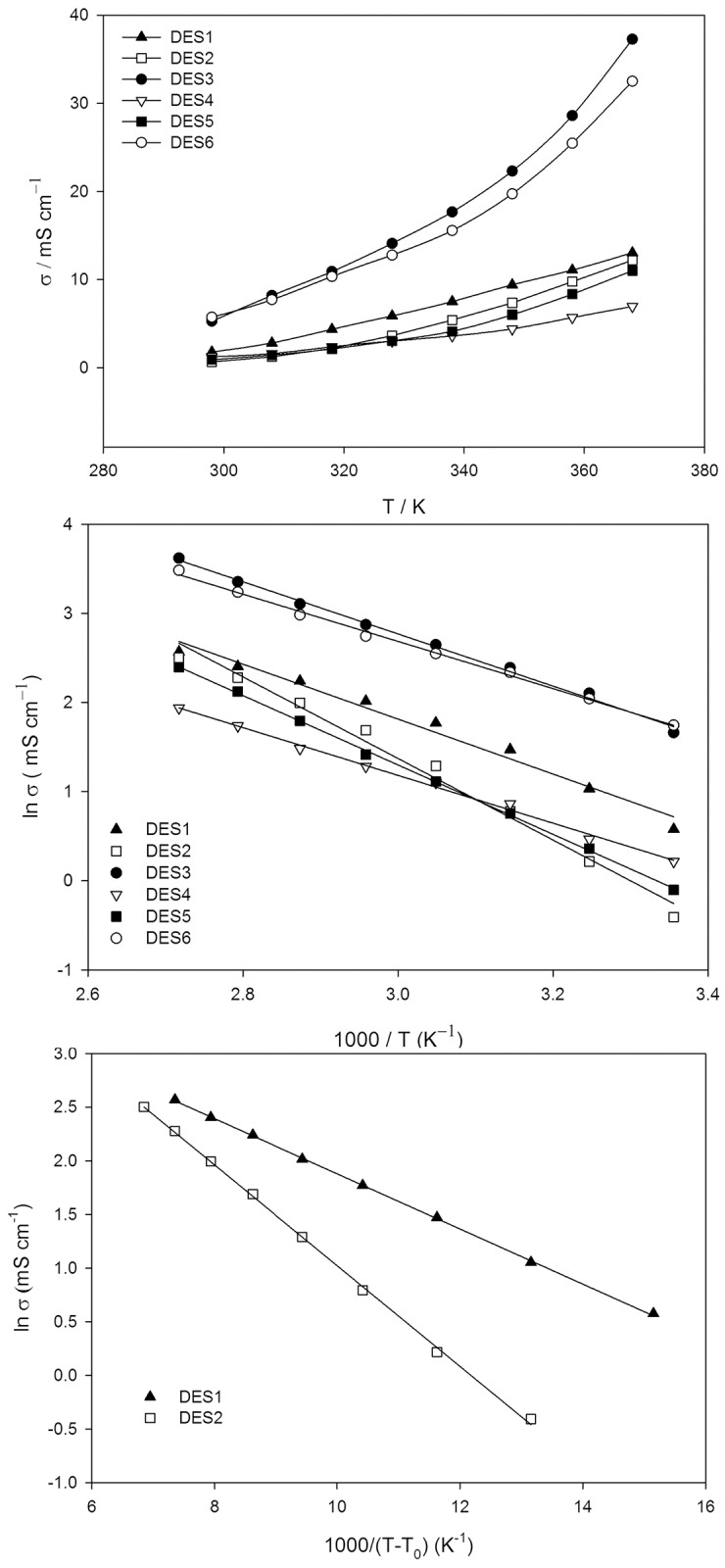
(a) Dependence of specific conductivity (*σ*) on temperature for the DESs; (b) Arrhenius plot of specific conductivity (*σ*) for the polyol based DESs; and (c) VTF plot of ionic conducticity for DES1 and DES2.

Fig 3(a) illustrates the temperature dependence of ionic conductivity (the most important property of electrolyte materials) for the DESs. Conductivity of DESs generally increase significantly as the temperature rises due to the ions moving faster at higher temperatures as a consequence of lower viscosities (of the neat DESs). The conductivity of the salts—ethylene glycol DESs (DES3, DES6)–show higher values in comparison to other polyol based DESs. Considering the hole theory for transport in molten salts, an Arrhenius equation (Eq (3)) was obtained for the temperature-dependence of the electrical conductivity (σ), which can be written as [52,53]:

$$\text{ln}\sigma =\text{ln}\sigma_{\text{0}}\text{+}\frac{{\text{E}}_{\sigma }}{\text{RT}}$$ (3)

Where *T* is the absolute temperature, *σ*_*0*_ is a constant and *E*_*σ*_ is the activation energy for conduction. Consequently, from Eq (3), *E*_*σ*_, *σ*_*0*_ and sums of square errors are tabulated in Table 2. Fig 3(b) reveals that in the case of the studied DESs, a linear relationship exists between T^-1^ and ln *σ* except DES1 and DES2, as predicted by the Arrhenius equation. The relation between ln(*σ*) and 1/*T* are well explained by a linear model with a correlation coefficient, R^2^, of more than 0.99 which indicates that the variation of electrical conductivity with temperature follows an Arrhenius model. However, the curvature of ln(*σ*) as a function of (1/*T*) in Fig 3(b), for DES1 and DES2 exhibit non-linear trends. Hence, for these two particular DESs, the Vogel–Tamman–Fulcher (VTF) was used to determine the temperature dependence of conductivity according to the following equation [54]:

$$\sigma =\sigma_{0}\;\text{exp}\left[\frac{-{\text{B}}_{\sigma }}{\text{T}-{\text{T}}_{\text{0}}}\right]$$ (4)

Where *σ*_*0*_, *T*_*0*_ and B_*σ*_ are fitting parameters. In Fig 3(c), the variation of ln (*σ*) *versus* 1/(*T*-*T*_*0*_) is plotted. The best fit values for *σ*_*0*_ (mS cm^-1^), B_*σ*_ (K) and *T*_*0*_ (K) are given in Table 3. *T*_*0*_ is supposed to have a close relationship with glass-transition temperature, and *σ*_*0*_ and B_*σ*_ are usually associated with several carrier ions and activation energies, respectively [55].

**Table 3:** The VTF equation parameters of the ionic conductivity for DES1 and DES2.

DESs	*T*_*0*_/ K	*σ*_*0*_ /mS cm^-1^	B_*σ*_ /(K)	*R* ^*2*^ ^a^
DES1	232	78	249	0.9994
DES2	212	435	557	0.9987

^a^ Correlation Coefficient

From the measured ionic conductivity *σ* (S m^-1^), the values of the molar conductivity *Λ* (m^2^ S mol^−1^) were calculated using *Λ* = (*M σ*) /*ρ*, where *M* and *ρ* are the respective equivalent weight and density of the DES. Solvent-free ionic liquids usually can be well illustrated by correlating the molar conductivity with temperature-dependent fluidity according to the modified Walden’s rule [56] using a qualitative approach [57]:

$$\Lambda \eta^{\alpha }=\;C$$ (5)

**Fig 4:**
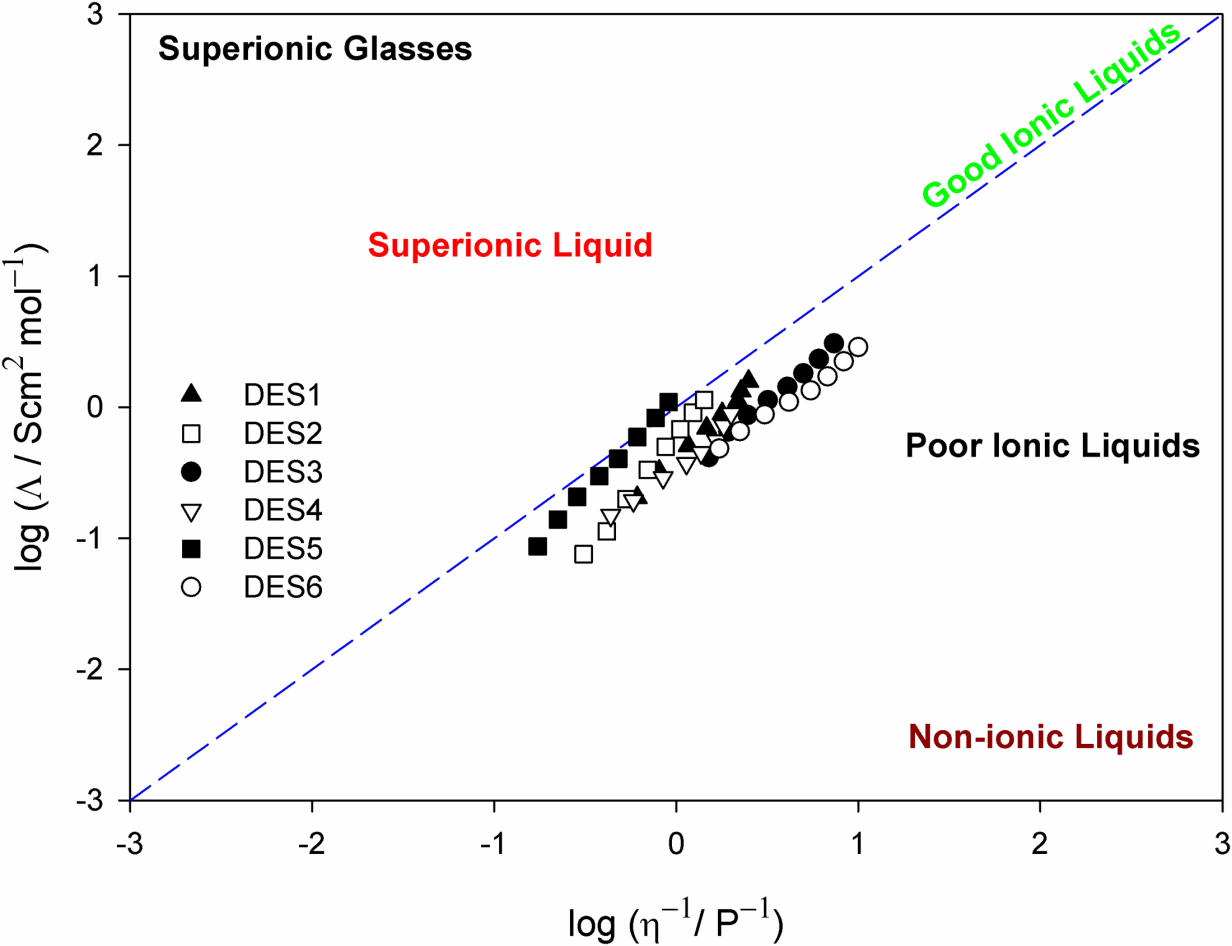
Walden plots for the polyol based DESs, where *Λ* is the molar conductivity and *η* ^-1^ is the fluidity. The dotted line indicates the data for a dilute aqueous KCl solution to fix the position of the ideal Walden line.

Where *α* is the slope of the line in the Walden plot, which reflects decoupling of the ions, and *C* (Walden product) is a temperature-dependent constant. This scheme is specifically appropriate in ILs [57], because it is a useful measure for examining ion pairing in electrolytes, and supplies the basis for comprehending the relationship between conductivity and viscosity. Fig 4 shows the Walden plot {ln(equivalent conductivity} versus ln{1/*η*)} over a temperature range of 298–368 K. The position of the ideal line is established using dilute aqueous KCl solutions in Fig 4, in which the system is known to be fully dissociated and to have ions of equal mobility. All DESs lie below the “ideal” Walden line. The deviations of the Walden plot of these DESs from the ideal line show an increased electrostatic interaction between the ammonium salts and the hydrogen bond donors.

### Electrochemical stability

**Fig 5:**
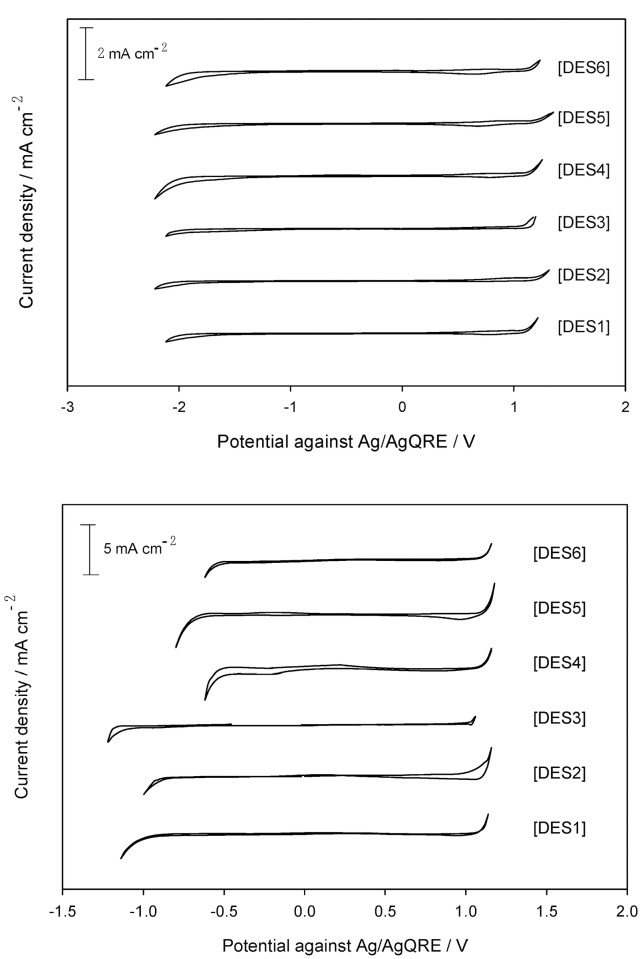
(a) Electrochemical stability of polyol-based DESs using GC working electrode; (b) Electrochemical stability obtained using Pt. microelectrode.

The electrochemical stability is one of the most important characteristics to be identified for electrolytes and solvents used in electrochemical applications. Electrochemical stability is dependent on the type of electrodes, the measurement situation, and the references employed. The arbitrary current cut-off used to define the onset of redox processes (typically between 0.1 and 1.0 mA cm^−2^) may not be strictly electrochemically determined. This difficulty is further combined in the case of ILs due to their sensitivity to air, water, and other impurities [58]. The cathodic stability of DESs is mainly determined by the potential at which the reduction of the cations (salts) takes place, while the anodic stability is measured where oxidation of the anions (HBDs) is expected to occur. For particular applications (e.g., super-capacitors), it is the overall potential window that matters, while in other applications, the actual anodic and cathodic limits associated to some reference is the restricting factor. The limiting reduction and oxidation potentials of the DESs are analyzed by performing cyclic voltammetry using a GC working electrode and a Pt. microelectrode at ambient temperature and at a scan rate of 0.1 V s^-1^, as shown in Fig 5(a) and 5(b) where the limiting current density reaches 0.2 mA cm^−2^.

The potential window of studied DESs, determined from the difference in potentials, is found to be similar on the GC electrode vs. Ag/AgQRE and has a wider potential range as compared to previously tested DESs [59]. In addition, the cathodic potential on the Pt. electrode (Fig 5a) is observed to decrease for the DESs in the order of **DES1 >DES2 >DES3** when using the choline chloride salt. However, for DESs made with the diethylenethanol ammonium chloride salt the cathodic potential values are similar. Such variations are not seen for the anodic potential [45].

### Temperature dependence of the voltammetric data for Fc/Fc^+^ and Cc^+^/Cc in DESs

Initially, in order to compare electrochemical parameters obtained in DESs as a function of temperature, it is essential to use either a reference electrode of a familiar potential against a standard reference electrode, or refer all data to a procedure whose reversible potential is presumed to be independent of the DESs. The oxidation of Fc is prevalently used to provide an internal potential scale standard in voltammetric studies [30–33]. Bond et al. have demonstrated that the reduction of Cc^+^ provides a broadly practical reference scale in both ILs and organic solvents [60,61]. In these DESs, Fc and Cc^+^ exhibit a reversible one-electron process.

$$\left[Fc{\left(C_{5}H_{5}\right)}_{2}\right]\Leftrightarrow {\left[Fc{\left(C_{5}H_{5}\right)}_{2}\right]}^{+}+e^{-}$$ (6)

$${\left[Co{\left(C_{5}H_{5}\right)}_{2}\right]}^{+}+e^{-}\Leftrightarrow [Co{\left(C_{5}H_{5}\right)}_{2}]$$ (7)

**Fig 6:**
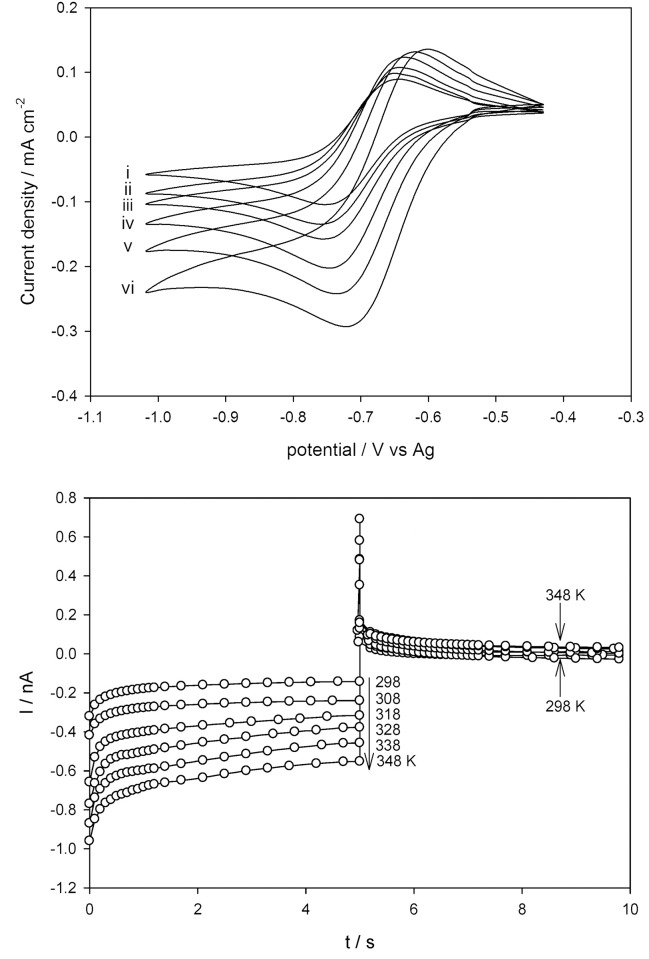
Cyclic voltammetry for the reduction of Cc^+^ in the polyol-based DESs at varying temperatures of: (i) 298 K, (ii) 308 K, (iii) 318 K, (iv) 328 K, (v) 338 K and (vi) 348 K, at 100 mV s^-1^. (b) Double potential step chronoamperometry measured on the same system across the Cc^+^/Cc at temperatures of 298, 308, 318, 338 and 348 K.

**Fig 7:**
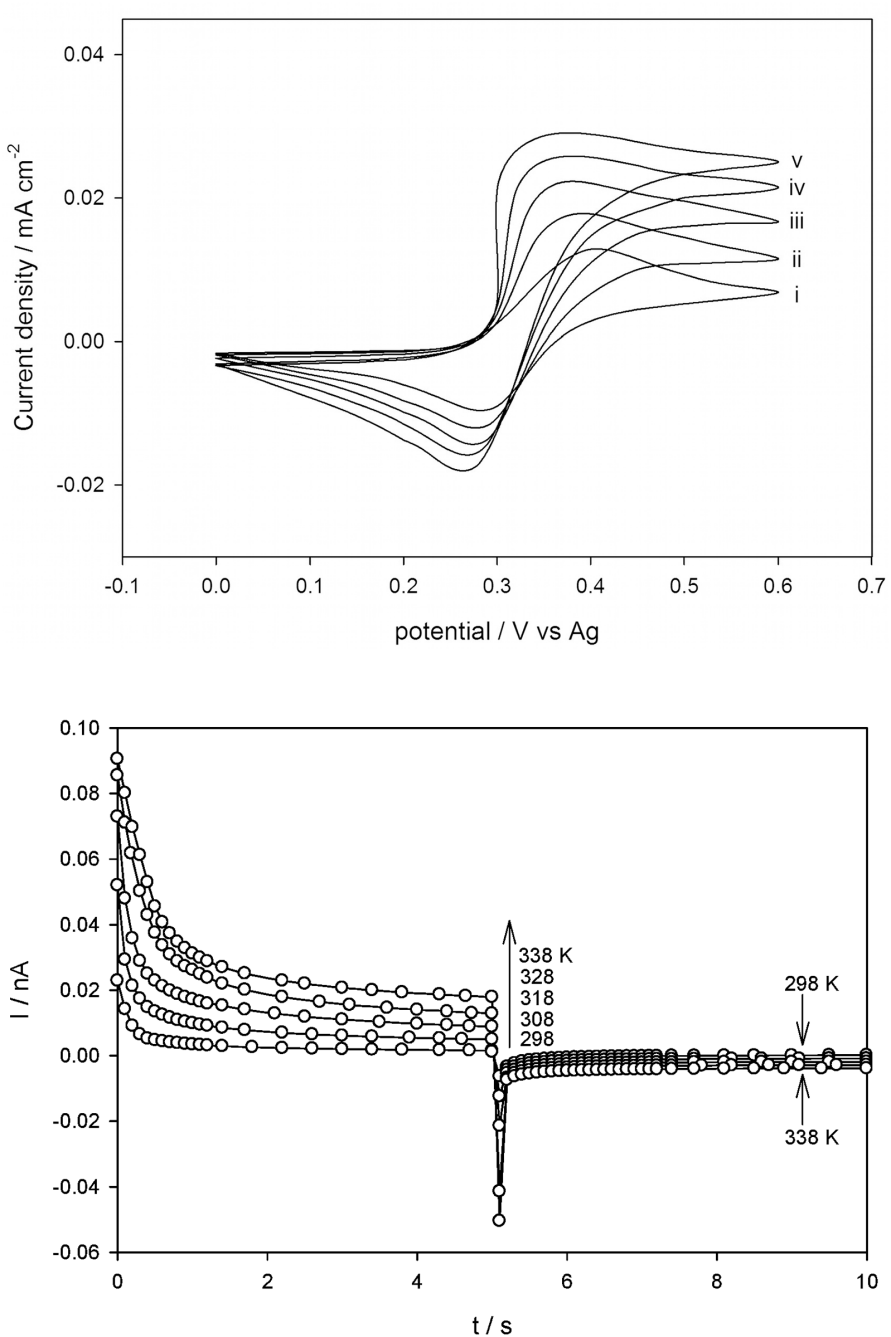
Cyclic voltammetry for the oxidation of Fc in polyol-based DESs at varying temperatures of: (i) 298 K, (ii) 308 K, (iii) 318 K, (iv) 328 K and (v) 338 K, at 100 mV s^-1^. (b) Double potential step chronoamperometry measured on the same system across the Fc/Fc^+^ at temperatures of 298, 308, 318 and 338 K.

Figs 6(a) and 7(a) indicate typical cyclic voltammograms for oxidation of Fc and reduction of Cc^+^ in DES1, respectively, which were obtained from individually prepared 10 mM solutions in the temperature range of 298–348 K. The voltammetric data of Fc and Cc^+^ is summarized in Table 4. Fc and Cc^+^ exhibited reversible reactions in all of the investigated DESs [45]. The anodic and cathodic peak currents had increased substantially with increasing temperature for oxidation of Fc and reduction of Cc^+^, respectively {Figs 6(a) and 7(a)}. The peak potential separation (*ΔE*_*p*_) was found to be in the range between 0.064–0.103 V for Fc and 0.073–0.103 V for Cc^+^ (Table 4). It was observed in all cases that *ΔE*_*p*_ decreased with increasing temperature and this at least partly reflected the faster electron kinetics. For DESs, the Δ*E*_*p*_ was found to be higher than the theoretical value (Δ*E*_*p*_ = 0.059 V), attributing to the effect of either slow heterogeneous electron transfer kinetics or the enhanced impact of Ohmic drop as was previously described by other researchers [32,34,36,41]. Reversible kinetics was assumed for both Fc and Cc^+^ following a similar presumption from work reported in the literature [38,40,62]. In addition, the half wave potential (*E*_*1/2*_) of the redox couples (vs. Ag/AgQRE) was determined according to the following equation

$$E_{1/2}=E_{PA}-\frac{\Delta E_{p}}{2}$$ (8)

Significant drifts in potential were initially observed for the AgQRE wire dipped directly in DESs, including electroactive compounds. However, separation of the Ag wire immersed in DESs from the bulk solution by means of a glass frit reduced this effect. The *E*_*1/2*_ data increased proportionately with temperature. Moreover, the *E*_*1/2*_ values changed significantly for different DESs at a given temperature. It was demonstrated that *E*_*1/2*_ shifted toward more negative potentials for the oxidation of Fc, and to more positive potentials for the reduction of Cc^+^ in DESs. The shift of *E*_*1/2*_ could be illustrated by the donor-acceptor Lewis-type interactions as discussed elsewhere [63].

**Table 4:** The electrochemical characteristics for Fc/Fc^+^ and Cc^+^/Cc with different operating temperature.

DESs	T/K	Fc/Fc^+^						Cc^+^/Cc					
		*j*_*pa*_/mA cm^-2^	*j*_*pc*_ /mA cm^-2^	*E*_*pa*_/V	*E*_*pc*_/V	*ΔE*_*p*_ /V	*E*_1/2_ /V	*j*_*pa*_/mA cm^-2^	*j*_*pc*_/mA cm^-2^	*E*_*pa*_/V	*E*_*pc*_ /V	*ΔE*_*p*_ /V	*E*_1/2_/V
DES1	298	0.012	-0.009	0.401	0.300	0.101	0.350	0.089	-0.104	-0.659	-0.759	0.100	-0.709
	308	0.017	-0.012	0.375	0.282	0.093	0.328	0.097	-0.129	-0.659	-0.758	0.099	-0.708
	318	0.021	-0.015	0.360	0.275	0.085	0.317	0.107	-0.154	-0.649	-0.749	0.100	-0.699
	328	0.025	-0.016	0.350	0.269	0.081	0.309	0.136	-0.201	-0.639	-0.738	0.099	-0.688
	338	0.028	-0.018	0.341	0. 265	0.076	0.303	0.145	-0.232	-0.619	-0.718	0.099	-0.668
DES2	298	0.029	-0.021	0.413	0.332	0.081	0.372	0.126	-0.143	-0.628	-0.723	0.095	-0.675
	308	0.035	-0.022	0.402	0.322	0.080	0.362	0.132	-0.162	-0.618	-0.710	0.092	-0.664
	318	0.040	-0.024	0.389	0.314	0.075	0.351	0.140	-0.187	-0.599	-0.685	0.086	-0.642
	328	0.043	-0.025	0.380	0.306	0.074	0.343	0.148	-0.231	-0.587	-0.666	0.079	-0.626
	338	0.047	-0.027	0.372	0.301	0.071	0.336	0.156	-0.259	-0.581	-0.654	0.073	-0.617
DES3	298	0.089	-0.075	0.425	0.322	0.103	0.373	0.079	-0.086	-0.672	-0.765	0.093	-0.718
	308	0.093	-0.076	0.417	0.321	0.096	0.369	0.087	-0.097	-0.665	-0.757	0.092	-0.711
	318	0.98	-0.079	0.411	0.319	0.092	0.365	0.096	-0.126	-0.654	-0.746	0.092	-0.700
	328	0.104	-0.083	0.400	0.314	0.086	0.357	0.111	-0.147	-0.643	-0.732	0.089	-0.687
	338	0.111	-0.086	0.388	0.306	0.082	0.347	0.124	-0.162	-0.631	-0.717	0.086	-0.674
DES4	298	0.021	-0.016	0.418	0.320	0.098	0.369	0.142	-0.171	-0.636	-0.738	0.102	-0.687
	308	0.026	-0.019	0.411	0.318	0.093	0.380	0.148	-0.183	-0.624	-0.722	0.098	-0.673
	318	0.031	-0.021	0.401	0.316	0.085	0.373	0.153	-0.192	-0.615	-0.711	0.096	-0.663
	328	0.035	-0.024	0.393	0.313	0.080	0.362	0.158	-0.221	-0.602	-0.694	0.092	-0.648
	338	0.038	-0.027	0.385	0.311	0.074	0.358	0.162	-0.245	-0.586	-0.673	0.087	-0.629
DES5	298	0.054	-0.045	0.421	0.334	0.087	0.377	0.107	-0.125	-0.661	-0.757	0.096	-0.709
	308	0.062	-0.048	0.416	0.330	0.086	0.373	0.116	-0.138	-0.653	-0.747	0.094	-0.700
	318	0.067	-0.052	0.406	0.323	0.083	0.364	0.124	-0.159	-0.642	-0.732	0.090	-0.687
	328	0.075	-0.055	0.395	0.313	0.082	0.354	0.132	-0.182	-0.633	-0.717	0.084	-0.675
	338	0.081	-0.059	0.388	0.309	0.079	0.348	0.141	-0.218	-0.618	-0.698	0.080	-0.658
DES6	298	0.064	0.059	0.434	0.345	0.089	0.389	0.091	-0.118	-0.652	-0.753	0.101	-0.702
	308	0.071	0.061	0.421	0.339	0.082	0.380	0.096	-0.129	-0.648	-0.746	0.098	-0.697
	318	0.079	0.063	0.411	0.336	0.075	0.373	0.098	-0.138	-0.640	-0.733	0.093	-0.686
	328	0.088	0.066	0.398	0.327	0.071	0.362	0.103	-0.156	-0.635	-0.725	0.090	-0.680
	338	0.096	0.069	0.390	0.326	0.064	0.358	0.105	-0.188	-0.628	-0.716	0.088	-0.672

### Calculation of Diffusional Activation Energies for Fc and Cc^+^

The diffusion coefficient, *D*, of both the Fc/Fc^+^ and Cc^+^/Cc redox couples in DESs at different temperatures were determined through analysis of double chronoamperometric measurements conducted at Pt. microelectrodes. The technique was undertaken using a sample time of 0.01 s. After pre-treatment by holding the potential at a point corresponding to zero Faradaic current for 20 s, the potential was stepped from 0 to +0.60 V (oxidation of Fc to Fc^+^) and -0.40 to -1 V (reduction of Cc^+^ to Cc), and the current was calculated for 5 s. The potential was then stepped back to 0 (reduction of Fc^+^ to Fc) and -0.40 V (oxidation of Cc to Cc^+^), and the current response was calculated for a further 5 s. The nonlinear curve fitting function in Origin 7.0 (MicroCal Software Inc.) following the approximation made by Shoup and Szabo [64] was used to fit the first potential step experimental data. The equations used in this approximation {Eqs (9)–(11)} describing the current response to within 0.6% over the entire time range is given below:

$$I=-4nFDcr_{d}f(\tau )$$ (9)

$$f(\tau )=0.7854+0.8863\tau^{-1/2}+0.2146\text{exp}(-0.7823\tau^{-1/2})$$ (10)

$$\tau =\frac{4Dt}{r_{d}^{2}}$$ (11)

Here, *r*_*d*_ represents the radius of the microdisk electrode, *D* is the diffusion coefficient, *F* is the Faraday constant, *c* is the bulk concentration of the electro-active species, *n* is the number of electrons transferred, and *t* is the time. Figs 6(b) and 7(b) show the best theoretical fit (O) to the experimental double potential step chronoamperograms (-) for the Fc/Fc^+^ and Cc^+^/Cc redox couples at 293, 298, 303, 308, 313, and 318 K in DES1. The limiting currents of the first step incline regularly and the trend of the second step becomes slightly less steep as the temperature increases in both Fc and Cc^+^. It is reasonably established that *D*_*Fc*_ and *D*_*Cc*_^*+*^ improves with increasing temperature in all six DESs (Table 5). The *D* of the electroactive species have been analyzed in terms of the Arrhenius exponential function of the temperature following the Eq (12).

$$D=D_{o}\text{exp}(\frac{-E_{D}}{RT})$$ (12)

Where *D*_*0*_ is a constant corresponding to the hypothetical diffusion coefficient at infinite temperature, and *E*_*D*_ is the diffusional activation energy of the electroactive species.

**Table 5:** Kinetic parameters and diffusion coefficients for Fc/Fc^+^ and Cc^+^/Cc in DESs at various temperatures.

DESs	*T*/K	*D*_*Fc*_/ cm^2^ s^-1^	*D*_*Cc*_^*+*^ / cm^2^ s^-1^	*k* ^*0*^_*Fc*_ / cm s^-1^	*k* ^*0*^_*Cc*_^*+*^ / cm s^-1^
DES1	298	1.71×10^−8^(±0.06)	0.65×10^−8^(±0.08)	2.49×10^−4^(±0.08)	1.96×10^−4^(±0.05)
	308	2.30×10^−8^(±0.03)	1.06×10^−8^(±0.07)	3.53×10^−4^(±0.07)	3.61×10^−4^(±0.06)
	318	2.93×10^−8^(±0.05)	1.52×10^−8^(±0.04)	4.65×10^−4^(±0.08)	5.80×10^−4^(±0.06)
	328	3.54×10^−8^(±0.08)	2.04×10^−8^(±0.06)	5.79×10^−4^(±0.09)	8.83×10^−4^(±0.05)
	338	4.23×10^−8^(±0.04)	2.63×10^−8^(±0.03)	7.12×10^−4^(±0.05)	1.25×10^−3^(±0.04)
DES2	298	4.02×10^−9^(±0.05)	3.26×10^−9^(±0.04)	2.20×10^−4^(±0.06)	1.92×10^−4^(±0.03)
	308	4.77×10^−9^(±0.05)	4.12×10^−9^(±0.07)	3.44×10^−4^(±0.07)	3.00×10^−4^(±0.08)
	318	5.46×10^−9^(±0.09)	4.98×10^−9^(±0.05)	4.73×10^−4^(±0.04)	4.16×10^−4^(±0.07)
	328	6.08×10^−9^(±0.06)	5.88×10^−9^(±0.08)	5.94×10^−4^(±0.04)	5.48×10^−4^(±0.08)
	338	6.86×10^−9^(±0.08)	6.88×10^−9^(±0.07)	7.89×10^−4^±(0.06)	7.23×10^−4^(±0.05)
DES3	298	3.10×10^−8^(±0.07)	2.22×10^−8^(±0.05)	3.08×10^−4^(±0.05)	2.68×10^−4^(±0.06)
	308	3.48×10^−8^(±0.02)	2.60×10^−8^(±0.09)	5.99×10^−4^(±0.05)	5.37×10^−4^(±0.07)
	318	3.94×10^−8^(±0.04)	3.06×10^−8^(±0.02)	1.16×10^−3^(±0.08)	1.07×10^−3^(±0.03)
	328	4.38×10^−8^(±0.05)	3.56×10^−8^(±0.06)	2.21×10^−3^(±0.06)	2.11×10^−3^(±0.06)
	338	4.92×10^−8^(±0.04)	4.11×10^−8^(±0.06)	4.73×10^−3^(±0.07)	3.96×10^−3^(±0.07)
DES4	298	4.41×10^−9^(±0.04)	3.60×10^−9^(±0.06)	2.31×10^−4^(±0.04)	2.15×10^−4^(±0.05)
	308	5.66×10^−9^(±0.09)	4.95×10^−9^(±0.07)	2.96×10^−4^(±0.07)	2.63×10^−4^(±0.06)
	318	6.85×10^−9^(±0.07)	6.37×10^−9^(±0.05)	3.59×10^−4^(±0.06)	3.13×10^−4^(±0.06)
	328	7.83×10^−9^(±0.04)	7.68×10^−9^(±0.08)	4.07×10^−4^(±0.05)	3.56×10^−4^(±0.08)
	338	9.00×10^−9^(±0.09)	9.22×10^−9^(±0.04)	4.61×10^−4^(±0.07)	4.01×10^−4^(±0.08)
DES5	298	3.23×10^−9^(±0.03)	3.33×10^−9^(±0.03)	1.68×10^−4^(±0.07)	1.63×10^−4^(±0.06)
	308	4.14×10^−9^(±0.06)	4.43×10^−9^(±0.08)	2.51×10^−4^(±0.04)	2.42×10^−4^(±0.04)
	318	5.37×10^−9^(±0.07)	5.53×10^−9^(±0.06)	3.54×10^−4^(±0.05)	3.29×10^−4^(±0.03)
	328	6.65×10^−9^(±0.02)	6.71×10^−9^(±0.08)	4.70×10^−4^(±0.08)	4.35×10^−4^(±0.07)
	338	8.26×10^−9^(±0.04)	7.83×10^−9^(±0.05)	6.32×10^−4^(±0.06)	5.47×10^−4^(±0.06)
DES6	298	3.29×10^−8^(±0.05)	2.89×10^−8^(±0.04)	5.44×10^−4^(±0.08)	4.35×10^−4^(±0.09)
	308	3.65×10^−8^(±0.04)	3.38×10^−8^(±0.04)	6.58×10^−4^(±0.07)	5.56×10^−4^(±0.08)
	318	3.99×10^−8^(±0.09)	3.84×10^−8^(±0.07)	7.69×10^−4^(±0.04)	6.85×10^−4^(±0.07)
	328	4.38×10^−8^(±0.06)	4.25×10^−8^(±0.05)	9.23×10^−4^(±0.05)	8.13×10^−4^(±0.06)
	338	4.82×10^−8^(±0.07)	4.84×10^−8^(±0.06)	1.14×10^−3^(±0.06)	1.02×10^−3^(±0.08)

Error bars calculated from the standard deviation from four experimental repetitions.

**Fig 8:**
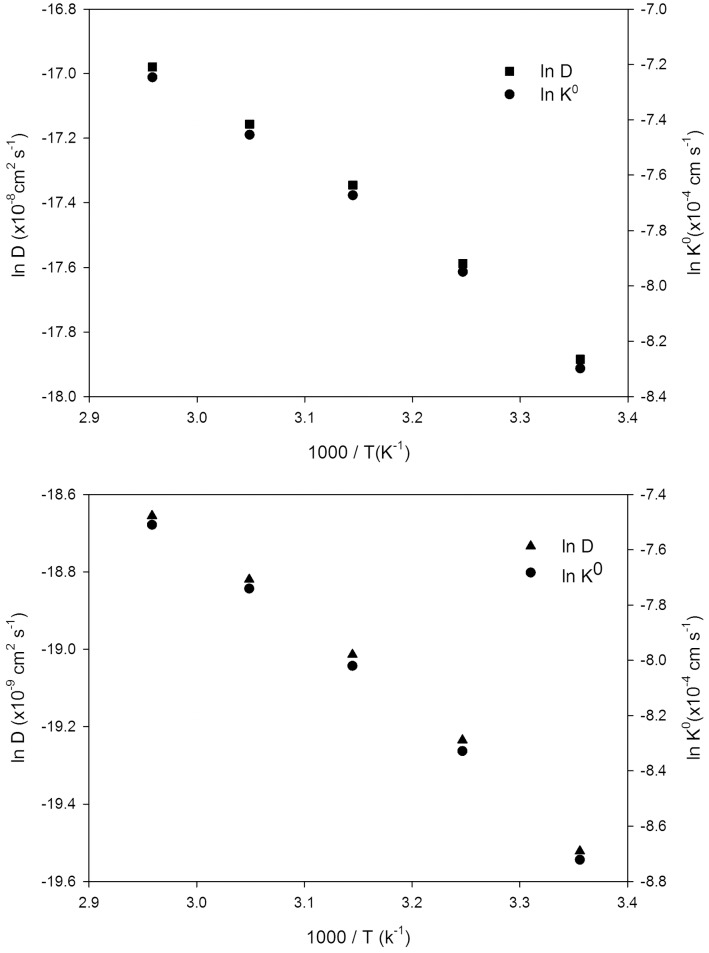
Dependence of diffusion and rate constant on temperature using Arrhenius rule for Fc (a) and Cc^+^ (b) redox couples.

Plot of ln*D* against 1/*T* resulted in a straight line, and from the slope the activation energy for diffusion, *E*_*D*_, was determined, as shown in Fig 8(a) and 8(b) (least-squares correlation coefficient, R^2^> 0.99 for Fc and Cc^+^). The calculated *E*_*D*_ for each sample is summarized in Table 6, which compares well with the value determined for the *E*_*η*_ in DESs and corresponds well to that observed in the literature for ILs [33]. The activation energies increased systematically with increasing viscosity in DESs. A slight deviation for activation energies of *D*_*Cc+*_ is observed, which may be due to impeded diffusion of the reduced species as a result of stronger solvation by the DES.

**Table 6:** Activation energies of the diffusion coefficients and rate constants for Fc/Fc^+^ and Cc^+^/Cc in studied DESs.

DESs	*E*_*D*_(Fc)/ kJ mol^-1^	*E*_*D*_(Cc^+^)/ kJ mol^-1^	*E*_*K*_^*0*^(Fc)/ kJ mol^-1^	*E*_*K*_^*0*^(Cc^+^)/ kJ mol^-1^
DES1	22.82	21.97	21.81	26.62
DES2	27.01	28.52	25.20	30.33
DES3	21.65	21.04	19.63	20.57
DES4	24.73	25.50	22.18	28.02
DES5	29.68	30.88	27.51	36.29
DES6	20.08	17.57	15.15	17.44

### Effect of temperature on kinetic electron transfer

The cyclic voltammetric data have been further analyzed for the evaluation of the heterogeneous electron transfer rate constant (*k* ^*0*^) of the Fc/Fc^+^ and Cc^+^/Cc redox couples according to the electrochemical absolute rate relation adapted from Nicholson’s method [65]. This is based on the variation of peak potential separation (*ΔE*_*p*_) between the forward and the reverse scans of the cyclic voltammogram for a simple one electron transfer process [41,66,67]. It is important to note that these values are only apparent, and are susceptible to errors resulting from charging currents and ohmic polarization, which can cause an overstatement of the rate constant. The rate constants of Fc and Cc^+^ increase with temperature, and Fig 8(a) and 8(b) display an Arrhenius relationship for the obtained *k* ^*0*^ values for Fc in DES1 and Cc^+^ for DES5, respectively. Nearly identical slopes are established for *D* and *k* ^*0*^ in the Arrhenius plots for selected DESs. From these plots, the activation energy of rate constants for Fc and Cc^+^ are determined, which are listed in Table 6.

## Conclusions

The effect of temperature on the measured physicochemical and electrochemical properties of the studied DESs has been discussed in detail. The activation energies for viscosity (*E*_*η*_) and conductivity (*E*_*σ*_) for each DES were calculated from the slopes of the Arrhenius plots. The temperature dependence of the measured conductivities did not exhibit linear behavior in the Arrhenius equation for DES1 and DES2, but it could be well described by the VTF model. The *D* and *k* ^*0*^ values tended to increase with the rise of temperature in DESs. The applicability of the Arrhenius law was verified by examining the temperature dependencies of *D* and *k* ^*0*^. The trends of electrochemically derived activation energy (*E*_*D*_) from chronoamperometric evaluations of DESs were found to be related with *E*_*η*_ and *E*_*σ*_ in the order of: **DES5 > DES2 > DES4 > DES1 > DES3 > DES6**. In addition, Δ*E*_*p*_ was reduced while *E*_*1/2*_ values shifted towards more negative potentials for oxidation of Fc and more positive potentials for reduction of Cc^+^ in DESs with the enhancement in temperature.

## References

[1] Zhao H, Baker GA. Ionic liquids and deep eutectic solvents for biodiesel synthesis: A review. Journal of Chemical Technology & Biotechnology 2012; 88: 3–12.

[2] Zhang Q, De Oliveira Vigier K, Royer S, Jerome F. Deep eutectic solvents: Syntheses, properties and applications. Chemical Society Reviews 2012; 41: 7108–7146. 10.1039/c2cs35178a 22806597

[3] Chakrabarti MH, Brandon NP, Hashim MA, Mjalli FS, AlNashef IM, Bahadori L, et al Cyclic voltammetry of iron (III) acetylacetonate in quaternary ammonium and phosphonium based deep eutectic solvents. International Journal of Electrochemical Science 2013; 8: 9652–9676.

[4] Rub C, Konig B. Low melting mixtures in organic synthesis—an alternative to ionic liquids? Green Chemistry 2012; 14: 2969–2982.

[5] Dai Y, van Spronsen J, Witkamp G-J, Verpoorte R, Choi YH. Natural deep eutectic solvents as new potential media for green technology. Analytica Chimica Acta 2013; 766: 61–68. 10.1016/j.aca.2012.12.019 23427801

[6] Chakrabarti M, Brandon N, Mjalli F, Bahadori L, Al Nashef I, Hashim MA, et al Cyclic voltammetry of metallic acetylacetonate salts in quaternary ammonium and phosphonium based deep eutectic solvents. Journal of Solution Chemistry 2013; 42: 2329–2341.

[7] Tang B, Row K. Recent developments in deep eutectic solvents in chemical sciences. Monatshefte fur Chemie—Chemical Monthly 2013; 144: 1427–1454.

[8] Abbott AP, Boothby D, Capper G, Davies DL, Rasheed RK. Deep eutectic solvents formed between choline chloride and carboxylic acids: Versatile alternatives to ionic liquids. Journal of the American Chemical Society 2004; 126: 9142–9147. 15264850 10.1021/ja048266j

[9] Smith EL, Abbott AP, Ryder KS. Deep eutectic solvents (DESs) and their applications. Chemical Review 2014; 114: 11060–11082. 10.1021/cr300162p 25300631

[10] Abbott AP, El Ttaib K, Ryder KS, Smith EL. Electrodeposition of nickel using eutectic based ionic liquids. Transactions of the Institute of Metal Finishing 2008; 86: 234–240.

[11] Chakrabarti MH, Manan NSA, Brandon NP, Maher RC, Mjalli FS, AlNashef IM, Hajimolana SA, Hashim MA, Hussain MA, Nir D. One-pot electrochemical gram-scale synthesis of graphene using deep eutectic solvents and acetonitrile. Chemical Engineering Journal 2015; 274: 213–223.

[12] Abbott AP, Ttaib KE, Frisch G, Ryder KS, Weston D. The electrodeposition of silver composites using deep eutectic solvents. Physical Chemistry Chemical Physics 2012; 14: 2443–2449. 10.1039/c2cp23712a 22249451

[13] Hu S, Zhang Z, Zhou Y, Song J, Fan H, Han B. Direct conversion of inulin to 5-hydroxymethylfurfural in biorenewable ionic liquids. Green Chemistry 2009; 11: 873–877.

[14] Serrano MC, Gutierrez MC, Jimenez R, Ferrer ML, Monte Fd. Synthesis of novel lidocaine-releasing poly(diol-co-citrate) elastomers by using deep eutectic solvents. Chemical Communications 2012; 48: 579–581. 10.1039/c1cc15284j 22109350

[15] Shahbaz K, Mjalli FS, H A. M, AlNashef IM. Using deep eutectic solvents for the removal of glycerol from palm oil-based biodiesel. Journal of Applied Sciences 2010; 10: 3349–3354.

[16] Abbott AP, Cullis PM, Gibson MJ, Harris RC, Raven E. Extraction of glycerol from biodiesel into a eutectic based ionic liquid. Green Chemistry 2007;9: 868–872.

[17] Hayyan M, Mjalli FS, Hashim MA, AlNashef IM. A novel technique for separating glycerine from palm oil-based biodiesel using ionic liquids. Fuel Processing Technology 2010; 91: 116–120.

[18] Abbott AP, Capper G, Davies DL, McKenzie KJ, Obi SU. Solubility of metal oxides in deep eutectic solvents based on choline chloride. Journal of Chemical & Engineering Data 2006; 51: 1280–1282.

[19] Abbott AP, Bell TJ, Handa S, Stoddart B. O-acetylation of cellulose and monosaccharides using a zinc based ionic liquid. Green Chemistry 2005; 7: 705–707.

[20] Zhang Z-H, Zhang X-N, Mo L-P, Li Y-X, Ma F-P. Catalyst-free synthesis of quinazoline derivatives using low melting sugar-urea-salt mixture as a solvent. Green Chemistry 2012; 14: 1502–1506.

[21] Dominguez de Maria P, Maugeri Z: Ionic liquids in biotransformations. From proof-of-concept to emerging deep-eutectic-solvents. Current Opinion in Chemical Biology; 15: 220–225. 10.1016/j.cbpa.2010.11.008 21112808

[22] Lindberg D, de la Fuente Revenga M, Widersten M. Deep eutectic solvents (dess) are viable cosolvents for enzyme-catalyzed epoxide hydrolysis. Journal of Biotechnology 2010; 147: 169–171. 10.1016/j.jbiotec.2010.04.011 20438773

[23] Gorke J, Srienc F, Kazlauskas R. Toward advanced ionic liquids. Polar, enzyme-friendly solvents for biocatalysis. Biotechnology and Bioprocess Engineering 2010; 15: 40–53. 10.1007/s12257-009-3079-z PMC8291719 34290544

[24] Carriazo D, Serrano MC, Gutierrez MC, Ferrer ML, del Monte F. Deep-eutectic solvents playing multiple roles in the synthesis of polymers and related materials. Chemical Society Reviews 2012; 41: 4996–5014. 10.1039/c2cs15353j 22695767

[25] Abbott AP, Capper G, Davies DL, Rasheed RK, Tambyrajah V. Novel solvent properties of choline chloride/urea mixtures. Chemical Communications 2003: 70–71. 10.1039/b210714g 12610970

[26] Maugeri Z, Dominguez de Maria P. Novel choline-chloride-based deep-eutectic-solvents with renewable hydrogen bond donors: Levulinic acid and sugar-based polyols. RSC Advances 2012; 2: 421–425.

[27] Ilgen F, Konig B. Organic reactions in low melting mixtures based on carbohydrates and l-carnitine-a comparison. Green Chemistry 2009; 11: 848–854.

[28] Abbott AP, Capper G, Gray S. Design of improved deep eutectic solvents using hole theory. ChemPhysChem 2006; 7: 803–806. 16596609 10.1002/cphc.200500489

[29] Kareem MA, Mjalli FS, Hashim MA, AlNashef IM: Phosphonium-based ionic liquids analogues and their physical properties. Journal of Chemical & Engineering Data 2010; 55: 4632–4637.

[30] Waligora L, Lewandowski A, Gritzner G. Electrochemical studies of four organometallic redox couples as possible reference redox systems in 1-ethyl-3-methylimidazolium tetrafluoroborate. Electrochimica Acta 2009; 54: 1414–1419.

[31] Hultgren VM, Mariotti AWA, Bond AM, Wedd AG. Reference potential calibration and voltammetry at macrodisk electrodes of metallocene derivatives in the ionic liquid [bmim][pf6]. Analytical Chemistry 2002; 74: 3151–3156. 12141676 10.1021/ac015729k

[32] Zhao C, Burrell G, Torriero AAJ, Separovic F, Dunlop NF, MacFarlane DR, et al Electrochemistry of room temperature protic ionic liquids. The Journal of Physical Chemistry B 2008; 112: 6923–6936. 10.1021/jp711804j 18489145

[33] Rogers EI, Silvester DS, Poole DL, Aldous L, Hardacre C, Compton RG. Voltammetric characterization of the ferrocene|ferrocenium and cobaltocenium|cobaltocene redox couples in RTILs. The Journal of Physical Chemistry C 2008; 112: 2729–2735.

[34] Sukardi SK, Zhang J, Burgar I, Horne MD, Hollenkamp AF, MacFarlane DR, et al Prospects for a widely applicable reference potential scale in ionic liquids based on ideal reversible reduction of the cobaltocenium cation. Electrochemistry Communications 2008; 10: 250–254.

[35] Torriero AAJ, Sunarso J, Howlett PC. Critical evaluation of reference systems for voltammetric measurements in ionic liquids. Electrochimica Acta 2012; 82: 60–68.

[36] Zhang J, Bond AM. Conditions required to achieve the apparent equivalence of adhered solid- and solution-phase voltammetry for ferrocene and other redox-active solids in ionic liquids. Analytical Chemistry 2003; 75: 2694–2702. 12948138 10.1021/ac026329f

[37] Crooks RM, Bard AJ. Electrochemistry in near-critical and supercritical fluids: Part VI. The electrochemistry of ferrocene and phenazine in acetonitrile between 25 and 300°c. Journal of Electroanalytical Chemistry and Interfacial Electrochemistry 1988; 243: 117–131.

[38] Tsierkezos N. Cyclic voltammetric studies of ferrocene in nonaqueous solvents in the temperature range from 248.15 to 298.15 k. Journal of Solution Chemistry 2007; 36: 289–302.

[39] Wang Y, Rogers EI, Compton RG. The measurement of the diffusion coefficients of ferrocene and ferrocenium and their temperature dependence in acetonitrile using double potential step microdisk electrode chronoamperometry. Journal of Electroanalytical Chemistry 2010; 648: 15–19.

[40] Matsumiya M, Terazono M, Tokuraku K. Temperature dependence of kinetics and diffusion coefficients for ferrocene/ferricenium in ammonium-imide ionic liquids. Electrochimica Acta 2006; 51: 1178–1183.

[41] Weaver JEF, Breadner D, Deng F, Ramjee B, Ragogna PJ, Murray RW. Electrochemistry of ferrocene-functionalized phosphonium ionic liquids. The Journal of Physical Chemistry C 2011; 115: 19379–19385.

[42] Barrosse-Antle LE, Hardacre C, Compton RG. Voltammetric currents in room temperature ionic liquids can reflect solutes other than the electroactive species and are influenced by carbon dioxide. The Journal of Physical Chemistry B 2009; 113: 2805–2809. 10.1021/jp810926u 19243203

[43] Guo Y, Kanakubo M, Kodama D, Nanjo H. Chronoamperometric determination of diffusion coefficients of ferrocene in ionic liquids mixed with co2 at high pressures. Journal of Electroanalytical Chemistry 2010; 639: 109–115.

[44] Taylor AW, Licence P, Abbott AP. Non-classical diffusion in ionic liquids. Physical Chemistry Chemical Physics 2011; 13, 10147–10154. 10.1039/c1cp20373h 21526251

[45] Bahadori L, Abdul Manan NS, Chakrabarti MH, Hashim MA, Mjalli FS, AlNashef IM, et al The electrochemical behaviour of ferrocene in deep eutectic solvents based on quaternary ammonium and phosphonium salts. Physical Chemistry Chemical Physics 2013; 15: 1707–1714. 10.1039/c2cp43077k 23247115

[46] Shahbaz K, Baroutian S, Mjalli FS, Hashim MA, AlNashef IM. Densities of ammonium and phosphonium based deep eutectic solvents: Prediction using artificial intelligence and group contribution techniques. Thermochimica Acta 2012; 527: 59–66.

[47] Leron RB, Soriano AN, Li M-H. Densities and refractive indices of the deep eutectic solvents (choline chloride+ethylene glycol or glycerol) and their aqueous mixtures at the temperature ranging from 298.15 to 333.15k. Journal of the Taiwan Institute of Chemical Engineers 2012; 43: 551–557.

[48] Leron RB, Wong DSH, Li M-H. Densities of a deep eutectic solvent based on choline chloride and glycerol and its aqueous mixtures at elevated pressures. Fluid Phase Equilibria 2012; 335: 32–38.

[49] Bandres I, Alcalde R, Lafuente C, Atilhan M, Aparicio S. On the viscosity of pyridinium based ionic liquids: An experimental and computational study. The Journal of Physical Chemistry B 2011; 115: 12499–12513. 10.1021/jp203433u 21942824

[50] Ciocirlan O, Iulian O, Croitoru O: Effect of temperature on the physico-chemical properties of three ionic liquids containing choline chloride. Rev Chim Bucharest 2010; 61: 721–723.

[51] Bonhote P, Dias A-P, Papageorgiou N, Kalyanasundaram K, Gratzel M. Hydrophobic, highly conductive ambient-temperature molten salts. Inorganic Chemistry 1996; 35: 1168–1178. 11666305 10.1021/ic951325x

[52] Vila J, Franjo C, Pico JM, Varela L, Cabeza O. Temperature behavior of the electrical conductivity of emim-based ionic liquids in liquid and solid states Portugaliae Electrochimica Acta 2007; 25: 163–172.

[53] Mamlouk M, Ocon P, Scott K.Preparation and characterization of polybenzimidzaole/diethylamine hydrogen sulphate for medium temperature proton exchange membrane fuel cells. Journal of Power Sources 2014; 245: 915–926.

[54] Vila J, Gines P, Pico JM, Franjo C, Jimenez E, Varela LM, Cabeza O. Temperature dependence of the electrical conductivity in EMIM-based ionic liquids: evidence of Vogel–Tamman–Fulcher behavior.Fluid Phase Equilibria 2006; 242: 141–146.

[55] Hayamizu K, Tsuzuki S, Seki S, Ohno Y, Miyashiro H, Kobayashi Y. Quaternary ammonium room-temperature ionic liquid including an oxygen atom in side chain/lithium salt binary electrolytes: ionic conductivity and 1H, 7Li, and 19F NMR studies on diffusion coefficients and local motions.The Journal of Physical Chemistry B 2008; 112:1189–1197 10.1021/jp077714h 18179199

[56] Bockris JOM, Reddy AKN. Modern Electrochemistry: Ionics. 2nd ed Plenum Press, New York, 1998.

[57] MacFarlane DR, Forsyth M, Izgorodina EI, Abbott AP, Annat G, Fraser K. On the concept of ionicity in ionic liquids. Physical Chemistry Chemical Physics 2009; 11: 4962–4967. 10.1039/b900201d 19562126

[58] Ong SP, Andreussi O, Wu Y, Marzari N, Ceder G. Electrochemical windows of room-temperature ionic liquids from molecular dynamics and density functional theory calculations. Chemistry of Materials 2011; 23: 2979–2986.

[59] Bahadori L, Chakrabarti MH, Mjalli FS, AlNashef IM, Manan NSA, Hashim MA. Physicochemical properties of ammonium-based deep eutectic solvents and their electrochemical evaluation using organometallic reference redox systems. Electrochimica Acta 2013; 113: 205–211.

[60] Shiddiky MJA, Torriero AAJ, Zhao C, Burgar I, Kennedy G, Bond AM. Nonadditivity of faradaic currents and modification of capacitance currents in the voltammetry of mixtures of ferrocene and the cobaltocenium cation in protic and aprotic ionic liquids. Journal of the American Chemical Society 2009; 131: 7976–7989. 10.1021/ja8092295 19507901

[61] Stojanovic RS, Bond AM. Examination of conditions under which the reduction of the cobaltocenium cation can be used as a standard voltammetric reference process in organic and aqueous solvents. Analytical Chemistry 1993; 65: 56–64.

[62] Torriero AAJ, Siriwardana AI, Bond AM, Burgar IM, Dunlop NF, Deacon GB, et al Physical and electrochemical properties of thioether-functionalized ionic liquids. The Journal of Physical Chemistry B 2009; 113: 11222–11231. 10.1021/jp9046769 19627093

[63] Keita B, Bouaziz D, Nadjo L. Solvent effects on the redox potentials of potassium 12tungstosilicate and 18 tungstodiphosphate. Journal of The Electrochemical Society 1988; 135: 87–91.

[64] Shoup D, Szabo A. Chronoamperometric current at finite disk electrodes. Journal of Electroanalytical Chemistry and Interfacial Electrochemistry 1982; 140: 237–245.

[65] Nicholson RS. Theory and application of cyclic voltammetry for measurement of electrode reaction kinetics. Analytical Chemistry 1965; 37: 1351–1355.

[66] Kim DY, Yang JC, Kim HW, Swain GM. Heterogeneous electron-transfer rate constants for ferrocene and ferrocene carboxylic acid at boron-doped diamond electrodes in a room temperature ionic liquid. Electrochimica Acta 2013; 94: 49–56.

[67] Pan Y, Cleland WE, Hussey CL. Heterogeneous electron transfer kinetics and diffusion of ferrocene/ferrocenium in bis(trifluoromethylsulfonyl)imide-based ionic liquids. Journal of the Electrochemical Society 2013; 159: F125–F133.

